# How Do Publicly Available Allergy-Specific Web-Based Training Programs Conform to the Established Criteria for the Reporting, Methods, and Content of Evidence-Based (Digital) Health Information and Education: Thematic Content Evaluation

**DOI:** 10.2196/12225

**Published:** 2019-10-24

**Authors:** Jonas Lander, Karin Drixler, Marie-Luise Dierks, Eva Maria Bitzer

**Affiliations:** 1 Institute for Epidemiology, Social Medicine and Health Systems Research Hannover Medical School Hannover Germany; 2 Department of Public Health and Health Education Freiburg University of Education Freiburg Germany

**Keywords:** allergy, asthma, health communication, health education, health information systems, evidence-based practice

## Abstract

**Background:**

Allergic diseases, such as allergic asthma, rhinitis, and atopic eczema, are widespread, and they are a considerable burden on the health care system. For patients and health care professionals, Web-based training programs may be helpful to foster self-management and provide allergy-specific information, given, for instance, their good accessibility.

**Objective:**

This study aimed to assess an exploratory sample of publicly available allergy-specific Web-based training programs—that is, interactive, feedback-oriented Web-based training platforms promoting health behavior change and improvement of personal skills—with regard to (1) general characteristics, aims, and target groups and (2) the extent to which these tools meet established criteria for the reporting, methods, and content of evidence-based (digital) health information and education.

**Methods:**

Web-based training programs were identified via an initial Google search and a search of English and German language websites of medical and public health services, such as the European Centre for Allergy Research Foundation (German), Asthma UK, and Anaphylaxis Canada. We developed a checklist from (1) established guidelines for Web-based health information (eg, the Journal of the American Medical Association benchmarks, DISCERN criteria, and Health On the Net code) and (2) a database search of related studies. The checklist contained 44 items covering 11 domains in 3 areas: (1) content (completeness, transparency, and evidence), (2) structure (data safety and qualification of trainers and authors), and (3) impact (effectiveness, user perspective, and integration into health care). We rated the Web-based training programs as completely, partly, or not satisfying each checklist item and calculated overall and domain-specific scores for each Web-based training program using SPSS 23.0 (SPSS Inc).

**Results:**

The 15 identified Web-based training programs covered an average of 37% of the items (score 33 out of 88). A total of 7 Web-based training programs covered more than 40% (35/88; maximum: 49%; 43/88). A total of 5 covered 30% (26/88) to 40% (35/88) of all rated items and the rest covered fewer (n=3; lowest score 24%; 21/88). Items relating to intervention (58%; 10/18), content (49%; 9/18), and data safety (60%; 1/2) were more often considered, as opposed to user safety (10%; 0.4/4), qualification of staff (10%; 0.8/8), effectiveness (16%; 0.4/2), and user perspective (45%; 5/12). In addition, in 13 of 15 Web-based training programs, a minimum of 3 domains were not covered at all. Regarding evidence-based content, 46% of all Web-based training programs (7/15) scored on use of scientific research, 53% on regular information update (8/15), and 33% on provision of references (5/15). None of 15 provided details on the quality of references or the strength of evidence.

**Conclusions:**

English and German language allergy-specific Web-based training programs, addressing lay audiences and health care professionals, conform only partly to established criteria for the reporting, methods, and content of evidence-based (digital) health information and education. Particularly, well-conducted studies on their effectiveness are missing.

## Introduction

### Background

The number of people affected by allergies and asthma varies around the world, but the prevalence of allergic diseases is high, particularly of allergic asthma, rhinitis, and atopic eczema [1-5]. Allergy-specific Web-based health information (WHI), as well as Web-based training programs (WTPs), can be a vital source of help for patients and health care professionals (HCPs). People affected by allergies and asthma, particularly those with mild-to-moderate symptoms, may not regularly see a physician but rely on self-treatment. This includes, for instance, nonprescription medicines, reading information on the Web, or even simply trying to get through the allergy season without help. Here, WHI or WTPs may be an alternative to *doing nothing* or relying only on one’s own knowledge and skills. Previous research has outlined the effectiveness of various measures [6,7]. For HCPs, allergy-specific WHI and/or WTPs might be relevant, given a need for continuous medical education and for support of their patient’s self-management skills [8]. We consider WTPs to comprise Web-based offers that go beyond mere provision of information, providing feedback and interactive learning opportunities promoting health behavior change and improvement of personal skills, without human interaction. We distinguish WTPs from services dedicated to Web-based treatment or counseling and from apps designed for digital mobile devices, such as tablets or phones [9-12]. A WTP works on desktop computers and mobile devices, but it will not need features specific to the mobile device, such as sensors or location awareness.

An allergy-specific WTP may assess patients’ current symptom avoidance practices during the allergy *season* and then give feedback on the effectiveness of that approach (feedback). If a patient is thereby encouraged to apply more effective approaches, this also improves self-management (personal skills). WTPs can also provide a diary for daily recording of symptoms medication use, which may then be shared electronically with a doctor or during a Web-based consultation (interactive learning). For professionals, a WTP could provide fictional cases of patients with allergic symptoms and guide them through the correct assessment and treatment (strengthen treatment skills and care practice). Although WTPs may be promising in general, previous research has highlighted a range of respective challenges in particular: limited abilities to access, understanding and applying health information (health literacy), poor-quality information and sources, use of jargon, inaccuracy, information overload, and a lack of universal requirements regarding content and methods [13-21]. By *quality*, we refer to the extent to which allergy-specific WTPs conform to established criteria on presentation of health information and its application to health care practice. Numerous initiatives have proposed criteria to ensure high-quality WHI (and hence WTPs), the most prominent being the *Journal of the American Medical Association* (JAMA) benchmarks [22], Health On the Net (HON) code [23], and the DISCERN criteria [24]. Yet, because of the sheer amount and variety of WHI, there is no one tool for evaluation or use of quality criteria that covers the entire spectrum of information sources [16]. The quality of intervention descriptions has also been criticized as making it difficult to build on or replicate available learning sources [25].

### Objectives

We are not currently aware of any overview of the quality of allergy-specific WTPs or the use of quality criteria by Web-based services intended for allergy sufferers or HCPs. A few studies have examined the effectiveness of digital asthma self-management [26,27], the impact of Web-based peer support for children with asthma [28], and Web-based support pilot studies [29]. Given this background, the objective of our study was to analyze an exploratory sample of publicly available, free-of-charge allergy-specific WTPs. Specifically, we analyzed (1) the general characteristics, aims, and target groups of WTPs, (2) the evidence base underlying WTPs’ content, and (3) the degree to which WTPs account for criteria on the reporting, methods, and content of (digital) health information (see also population, intervention, comparison, outcome table in Multimedia Appendix 1). As aspects, such as structure, quality of information, and evidence base, are relevant independent of the WTPs’ target group, we did not limit our analysis to either lay people or HCPs.

## Methods

### Search Strategy and Selection of Web-Based Training Programs for Assessment

**Figure 1:**
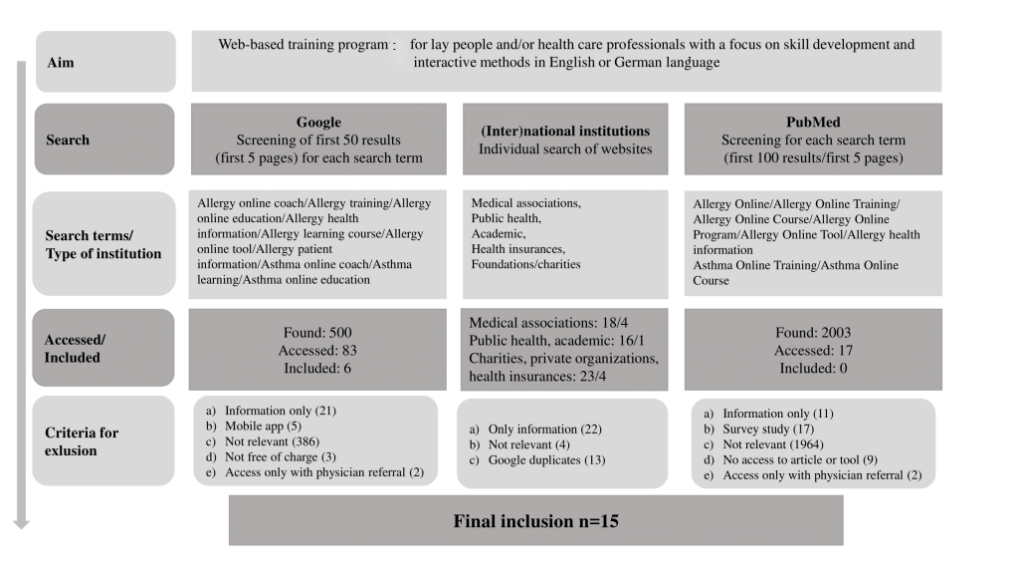
Search Strategy. WTP: Web-based training program; HCP: health care professional.

We conducted a Google search to retrieve and select relevant WTPs. As this is an exploratory study, we constrained the search to WTPs in English and German, that is, the languages spoken by our research team. We used a public search engine, as the Web-based sources need to be available to lay people, rather than being available only in a scientific database intended for experts. We added a search in PubMed to check for potentially relevant WTPs not found in Google and for scientific evaluations of WTPs. For the search, terms were combined with regard to (1) indication/disease and (2) the diverse types of available Web-based sources (Figure 1).

In addition, we searched country-specific websites of institutions that provide general health information or specialize in allergies and atopic diseases: medical associations, health insurers, state health services, and official Public Health information portals. This included the National Health Service, Allergy UK (United Kingdom), the American Academy of Allergy (United States), Allergy Aware Canada (Canada), Australasian Society of Clinical Immunology and Allergy (Australia), the German Medical Association, the European Centre for Allergy Research Foundation (Germany), the European Academy of Allergy and Clinical Immunology (Switzerland), and the World Allergy Organization (United States). From these sources, we included tools that provided allergy- and/or asthma-specific training for lay people and HCPs (medical doctors, nurses, school staff, and pharmacists). We also categorized retrieved sources as either (1) informative websites, (2) apps, or (3) WTPs. Next, we selected only services and tools of type (3), according to the abovementioned description of WTPs, to limit the analysis to the more comprehensive, interactive learning approaches. We ended the search after no new, additional WTPs were found by different searches using the same search strategies. Criteria for exclusion were as follows: not free of charge, available only by physician referral, and not yet or no longer publicly available.

### Checklist: Development and Application

To assess the selected WTPs, we first conducted a PubMed search for standards, guidance, and tools to develop, report, and/or critically appraise WHI and WTP. We then summarized the criteria from different sources [9,22,23,25,30] and adapted the description and wording of each criterion into an assessment item (Multimedia Appendix 2). The summary checklist resulted in a list of 44 items, subdivided into 11 domains (Textbox 1).

For each WTP, 3 researchers read and worked through the respective program and extracted relevant text passages independently. The extracted material was given one of the following ratings:

- Yes: criterion satisfied according to the available information
- Partly: criterion satisfied to some extent
- No (not done or not stated): criterion not satisfied or no information about whether the aspect/content relevant to the respective criterion was not considered or simply not stated
- Not included: the WTP’s design does not address the criterion, although it seems relevant

After initial assessment, missing information and unclear ratings were discussed among the 3 researchers to reach agreement. Finally, we assigned a score for each rating (yes=2, partly=1, no=0, and not part of WTP=–1) and calculated per-domain and overall scores for each WTP, using SPSS 23. For instance, the category ‘indication’ includes 9 criteria, hence a maximum per-domain score of 18 can be given.

**List of quality criteria for Web-based training programs, adapted from studies.**

Indication

- The symptoms addressed by the program are described
- The levels of severity of the allergy, with which the program is supposed to help, are described

Intervention

- Full provider contact details are given
- The program type (self-help, coaching, chat, etc) is described
- The description of the type of program is transparent and freely accessible
- Rationales and aims are described
- The program is described separately for other target groups (who may also be interested in the content), either for lay people or professionals
- A minimum/maximum usage time is mentioned
- A certain usage time is recommended
- The recommended usage time is supported by evidence
- Alternatives for using this particular program are mentioned

Content

- The information has been researched scientifically and systematically
- The information is up to date
- The information is updated regularly according to most recent available knowledge
- Transparent sources/references are provided
- The content of the information is formulated neutrally and factually
- The information/content mentions potential uncertainties and risks
- Transparent information regarding financing and conflicts of interest are provided
- Potential usage/user differences because of age or sex are mentioned
- The content is differentiated for/adapted to different target groups

Safety

- Potential unintended effects of using the program are described
- The program describes what happens in case of an unintended effect

Qualification

- Users can contact an expert
- The qualification of the expert is described (if part of intervention)
- Experts that can be contacted use an intervention manual
- Experts are being supervised

Effectiveness

- The effectiveness of the program is assessed (via a scientific evaluation)

User perspective

- The program is accessible (eg, by hearing- or vision-impaired users)
- The program is free of charge
- The program is available in different languages
- Completion/termination rates are mentioned
- User satisfaction with the program is assessed
- The success of the program is assessed (have the users completed the modules successfully)

Integration into care

- User behavior is followed up
- Users can communicate with other users and/or other professionals

Legal aspects

- It is stated who is liable in case of mistakes and adverse effects

Data safety

- It is clearly mentioned that data safety is ensured by the provider
- The user can register anonymously
- A description of how user data are stored is given
- It is mentioned for how long the data are stored
- The user can ask the provider to delete personal data
- The program requires specific software (eg, operating system or browser)

Advertisement

- The program includes open/direct advertisement
- The program includes indirect advertisement

## Results

### General Characteristics

The search found 171 potentially relevant (and accessed) allergy-specific WTPs, with 15 remaining after applying exclusion criteria (Figure 1). Regarding those WTPs found via PubMed, 3 WTPs required a fee, and 4 WTPs were only available by physician referral. A total of 5 WTPs were not publicly available, as they were part of a (closed) intervention study [28,31-34]. Another 3 WTPs could not be found on the internet, or they were no longer available. Providers of the 15 WTPs included in this analysis were based in the United Kingdom (n=1), Germany (n=4), the United States (n=3), Australia (n=4), Canada (n=2), and one was not country specific. WTPs were run by (1) academic institutions (n=1), nonprofit foundations/charities (n=7), medical societies (n=3), and umbrella organizations thereof (n=2), publishing houses (n=1), and a pharmaceutical training portal (n=1). A total of 14 of the 15 programs required registration and a user account for access. Of the included WTPs, 53% (n=8) mentioned (only) 1 subject, for example, asthma, anaphylaxis, pollen allergy, or simply *allergy* without a focus. A total of 33% (n=5) of the WTPs combined 2 subjects, for example, allergy and anaphylaxis, or atopic eczema and food allergies. Except for the combination of a specific allergy with anaphylaxis, WTPs mentioned no reasons for combining subjects. The 2 programs with 3 or more subjects aimed to provide a holistic overview of the various types of allergies and how to deal with them. WTPs varied widely in their style of presentation (Table 1).

Some (n=5) referred to an *interactive online course* or *interactive learning module*; others described their program as “online” or “e-learning” modules (n=4). Nevertheless, others simply referred to an *education course*, *active learning*, or *individual learning* modules and course (n=4), whereas a final group focused on describing the format: *scientific animation* and *multimedia tool* (n=2).

With regard to the precise didactic methods, 60% (n=9) of the WTPs applied 4 or more methods. A total of 1 WTP, for instance, combined visualized written information, animations, videos, written instructions, case studies, a survey, a quiz, and a feedback form. Another WTP applied preknowledge questions, learning modules (short written information), short video, multiple-choice questions for self-assessment, and a course certificate for download after completion. A total of 2 WTPs used 3 different methods, and 4 WTPs applied only 1 (n=1) or 2 (n=3) methods, for example, fictional characters telling their disease story and interacting with the user via questions. The WTPs did not explain why particular methods were chosen, their didactic approach/model, or the didactic advantage of their chosen methods. The WTPs addressed 3 broadly distinguishable audiences: (1) lay people (ie, allergy sufferers), (2) HCPs (including physicians), and (3) people who are not medical experts but work in the public sector, that is, teachers and other school staff. The latter were mentioned twice as the sole target group. A total of 1 WTP addressed only lay people. A total of 7 WTPs were aimed at professionals, mostly referring to *doctors* and *professionals*. The other 5 WTPs addressed mixed target groups, that is, lay people and HCPs.

**Table 1:** General information on assessed Web-based training programs.

Name		Subjects	Country	Provider	Language	Aim	Methods
**Lay people only**							
	Anaphylaxis First Aid Training Community	Anaphylaxis	Australia	Australasian Society of Clinical Immunology and Allergy (medical society)	English	Provide ready access to reliable anaphylaxis education	Learning modules; Visualizations; Short video; Certificate; Evaluation
**Health care professionals only**							
	Allergic Rhinitis e-training	Allergic rhinitis	Australia	Australasian Society of Clinical Immunology and Allergy (medical society)	English	Provide ready access to reliable allergic rhinitis education	Introductory questions; Learning modules; Visualization; Short video; Multiple-choice self-control questions; Certificate
	Allergy and Anaphylaxis e-training for Health Care Professionals	Allergies; Anaphylaxis	Australia	Australasian Society of Clinical Immunology and Allergy (medical society)	English	Provide ready access to reliable anaphylaxis rhinitis education	Learning modules; Final assessment; Evaluation; Certificate
	Asthma and Allergic Rhinitis: World Allergy Organization Online Learning Series	Asthma; Allergic rhinitis	United States	World Allergy Organization (international umbrella organization of medical societies)	English	Provide a clear overview about asthma and allergic rhinitis	Presentation by speaker; Question and Answer session
	Asthma management and education course	Asthma	United States	Asthma and Allergy Foundation of America (foundation/nonprofit organization)	English	Educate health professionals about asthma management	Learning modules, including assessment; Evaluation
	e-learning hub	Asthma	Australia	Asthma Australia (foundation/nonprofit organization)	English	Guide professionals in asthma management and patient education	Audiovisual presentation; Certificate; Survey; Review questions
	National Asthma Council Australia Online Learning	Asthma	United States	National Asthma Council Australia (nonprofit charity)	English	Provide professionals with interaction learning on the latest information and resources on asthma	Learning modules; Presentation, including how-to videos; Introductory questions; Case studies
	Azerta	Allergies (various/general)	Germany	Azerta Apotheken Portal (pharmacy training portal)	German	Continuing education	Videos; “Coaching” for dealing with clients; Questionnaire (knowledge questions); Certificate
**Lay people and health care professionals**							
	Itchy Sneezy Wheezy	Asthma; Allergy; Allergic Rhinitis; Eczema	England	Imperial College London (lead) with partner organizations (academic)	English	Train and improve users’ knowledge and skills	Written information; Slides; Multimedia guide; Feedback form; Certificate
	Human-Biomonitoring	Environmental substances (main part); Allergies (subpart)	Germany	Allum/ Kinderumwelt gemeinnützige GmbH Osnabrück (umbrella organization of medical societies)	German; English	Provide knowledge and information interactively	Animation
	Allergyaware	Anaphylaxis	Canada	Food Allergy Canada (charitable organization)	English; French	Provide information and knowledge to improve behavior/skills for emergency situations	Visualized information; Animation; Videos; Instructions; Case studies; Survey; Quiz; Feedback form; Certificate
	Asthma Basics	Asthma	United States	American Lung Association (voluntary health organization/nonprofit organization)	English	Help people learn more about asthma	Introductory questionnaire; Interactive presentation; Repeat questions; Quiz; Evaluation; Certificate
	Lunge in Not	Asthma	Germany	Springer Medizin Verlag GmbH (publishing house)	German	Improve patient–physician communication	Use of fictional characters to visualize information; Storyline (scroll down)
**Others**							
	Back to School	Food allergies; Anaphylaxis	Canada	Food Allergy Research and Education (nonprofit organization)	English	Support school staff in the prevention and management of food allergies and anaphylaxis	Multiple choice; Warmup questions; Visualized information; Audio sections (information/facts); Final quiz; Certificate
	Bist du auch allergisch?	Neurodermatitis; Food allergies	Germany	European Centre for Allergy Research Foundation (nonprofit foundation)	German	Independent Web-based learning; knowledge acquisition; knowledge application	Learning modules/chapter; Assessment questions

### Indication and Intervention

In the domains *indication* (2 items) and *intervention* (9 items), almost all WTPs covered at least 50% of the total items, resulting in a mean score of 52% (sum score: 2.1/4) for indication and 58% (sum score: 10.4/18) for intervention (Table 2). For *indication*, all WTPs described the symptoms addressed as part of the content and learning modules, but none described the severity of symptoms that would or would not be addressed (although 1 WTP was rated *partly*).

With regard to the description of interventions, 8 of 15 WTPs covered more than half of the items for this category; 6 of those covered more than two-thirds. General aspects, such as rationales/aims (100%; 15/15), intervention type (86%; 13/15), minimum/maximum usage time (60%; 9/15), and provider details (66%; 10/15), were covered most often. More specific aspects, such as separate intervention descriptions according to the respective target group (13%; 2/15), alternatives to using the WTP (27%; 4/15), and evidence for the recommendation of a specific usage time (13%; 2/15), were addressed much less frequently. For example, 1 WTP stated “You can undertake this course at your own pace, but it is recommended that you complete the modules within a two-week period,” though without stating who exactly recommends this (see Multimedia Appendix 3). In total, items belonging to the domains indication (2) and intervention (9) were more often rated as fulfilled or partly fulfilled (61%, n=101 ratings) than not fulfilled (39%, n=64 ratings; Table 2).

**Table 2:** Per-domain and overall scores.

Name and WTP^a^		Score, n (%)											
		Indication	Intervention	Content	Safety	Qualification	Effect	User perspective	Care	Legal	Data safety	Advertisement	Total
**Lay people** **, n (%)**													
	WTP1	2 (50)	14 (78)	12 (67)	0 (0)	1 (12)	0 (0)	4 (33)	0 (0)	2 (100)	2 (17)	2 (50)	39 (44)
**Professionals, n (%)**													
	WTP2	2 (50)	8 (44)	11 (61)	0 (0)	1 (12)	0 (0)	5 (42)	0 (0)	2 (100)	2 (17)	2 (50)	33 (37)
	WTP3	2 (50)	12 (67)	10 (56)	0 (0)	1 (12)	2 (100)	6 (50)	0 (0)	2 (100)	2 (17)	2 (50)	38 (43)
	WTP4	2 (50)	8 (44)	5 (28)	0 (0)	2 (25)	0 (0)	4 (33)	0 (0)	0 (0)	0 (0)	2 (50)	23 (26)
	WTP5	2 (50)	12 (67)	4 (22)	0 (0)	1 (12)	0 (0)	8 (67)	0 (0)	0 (0)	0 (0)	0 (0)	27 (31)
	WTP6	2 (50)	13 (72)	11 (61)	0 (0)	0 (0)	0 (0)	6 (50)	0 (0)	0 (0)	2 (17)	2 (50)	36 (41)
	WTP7	2 (50)	14 (78)	12 (67)	0 (0)	2 (25)	0 (0)	4 (33)	0 (0)	2 (100)	5 (42)	0 (0)	41 (47)
	WTP8	2 (50)	9 (50)	7 (39)	0 (0)	0 (0)	0 (0)	4 (33)	0 (0)	2 (100)	6 (50)	2 (50)	32 (36)
**Lay people and professionals, n (%)**													
	WTP9	2 (50)	10 (56)	9 (50)	0 (0)	0 (0)	0 (0)	6 (50)	0 (0)	0 (0)	0 (0)	0 (0)	27 (31)
	WTP10	3 (75)	9 (50)	11 (61)	0 (0)	3 (37)	0 (0)	6 (50)	0 (0)	0 (0)	4 (33)	0 (0)	36 (41)
	WTP11	2 (50)	9 (50)	11 (61)	4 (100)	1 (12)	0 (0)	8 (67)	0 (0)	2 (100)	4 (33)	2 (50)	43 (49)
	WTP12	2 (50)	9 (50)	5 (28)	0 (0)	0 (0)	2 (100)	10 (83)	0 (0)	2 (100)	4 (33)	0 (0)	34 (39)
	WTP13	2 (50)	6 (33)	7 (39)	0 (0)	0 (0)	0 (0)	4 (33)	0 (0)	0 (0)	2 (17)	0 (0)	21 (24)
**Others n (%)**													
	WTP14	2 (50)	13 (72)	11 (61)	2 (50)	0 (0)	2 (100)	4 (33)	0 (0)	2 (100)	0 (0)	2 (50)	38 (43)
	WTP15	2 (50)	10 (56)	6 (33)	0 (0)	0 (0)	0 (0)	2 (16.7)	0 (0)	2 (100)	4 (33)	0 (0)	26 (29)
**Summary**													
	WTP, mean (SD)	2.1 (52)	10.4 (58)	8.8 (49)	0.4 (10)	0.8 (10)	0.4 (17)	5.4 (45)	0 (0)	1.2 (60)	2.5 (21)	1.1 (27)	32.9 (37)
	Maximum, n (%)	4 (100)	18 (100)	18 (100)	4 (100)	8 (100)	2 (100)	12 (100)	4 (100)	2 (100)	12 (100)	4 (100)	88 (100)

^a^WTP: Web-based training program.

### Content and Safety

At least half the items related to content (n=9) were covered by 9 of 15 WTPs, that is, a score of at least nine out of 18, the maximum covered being 67% (12 out of 18). Individual items often fulfilled were evidence-based content (n=10; yes=7; partly=3), provision of a date to indicate up to dateness (n=8), regular updates of the information (n=8), and neutral and factual content and language (n=15). Regarding the latter, this was rated as fulfilled when the information did not include obvious exaggerations or strongly positive/negative formulations, for example, regarding a treatment option. Furthermore, 6 of 15 WTPs covered fewer than 40% of items in this category, 3 of those covering only 20% to 30%. Items that were often not covered included the provision of full references for all content (partly=5; no=5), possible differences in how the service should be used because of age and/or sex (n=0), uncertainties and risks, for example, when following a specific recommendation or knowledge gaps (n=5; see Table 3 for examples), and funding and conflicts of interest (yes=5; partly=6).

Although references were provided by several WTPs (see above), none provided explicit descriptions of the quality of those references, the type and strength of evidence within these references, and how the references were used to develop content to cover the (full) spectrum of relevant, evidence- and/or expert-based issues regarding prevention, diagnosis, and treatment. Given the number of WTPs covering less than 40% of all content-related items, the mean score for this category was 49% (sum score 8.8 of 18). Of all ratings (n=135), 42% (n=75) of the ratings were positive (“yes”) and 44% (n=60) of the ratings were negative (“no” or “not addressed”); the rest were rated “partly” (14%).

**Table 3:** Rating summary.

Domain	Item, n	Total ratings, n	Yes, n (%)	Partly, n (%)	No, n (%)	Not part, n (%)
(1) Indication	2	30	15 (50)	0 (0)	13 (43)	2 (7)
(2) Intervention	9	135	70 (51.8)	16 (11.8)	39 (28.8)	10 (7.4)
(3) Content	9	135	56 (41.4)	19 (14.0)	41 (30.3)	19 (14.0)
(4) Safety	2	30	3 (10)	0 (0)	10 (33)	17 (57)
(5) Qualification of staff	4	60	3 (5)	6 (10)	28 (47)	23 (38)
(6) Effectiveness	1	15	3 (20)	0 (0)	12 (80)	0 (0)
(7) User perspective	6	90	40 (44)	1 (1)	49 (54)	0 (0)
(8) Integration into care	2	30	0 (0)	0 (0)	0 (0)	30 (100)
(9) Legal aspects	1	15	9 (60)	0 (0)	6 (40)	0 (0)
(10) Data safety	6	90	18 (20)	0 (0)	55 (61)	17 (19)
(11) Advertisement	2	30	8 (27)	0 (0)	22 (73)	0 (0)
Rating summary	44	660	225 (34.0)	43 (6.5)	275 (41.6)	118 (17.8)

Regarding user safety from potential adverse effects during or because of the provided content, many WTPs included only general information and learning material, which is unlikely to have serious effects. Therefore, *information about adverse effects* (n=8) and *system reaction in case of adverse events* (n=9) were often not addressed. Programs with more serious content, for example, instructions for using an anaphylaxis auto-injector, mostly did not provide respective information (n=4) or a description of what happens in case of an emergency/adverse reaction from the user (n=6). Only 2 of 15 WTPs addressed any safety-related items, resulting in a mean sum score of 10% (0.8 of 8).

### User Perspective and Qualification of Staff

Items in these domains were covered to a varying extent, resulting in a sum score of 5.4 out of 12 (45%) for user perspective and 0.8 out of 8 (10%) for qualification of staff. Regarding user perspective, 7 WTPs covered at least 50% of all items in this category (n=6); 1 WTP covered 83%. Of the remaining WTPs (n=8) with scores below 50%, 6 WTPs covered only 33%. Items that were addressed least often included WTPs being free of barriers; only 5 WTPs provided services for handicapped users, such as listening sessions, availability of different languages (n=5), none provided a description of completion rates. A total of 8 WTPs assessed user satisfaction, mostly via a survey after completion of the learning modules, and 7 assessed the learning outcomes and whether users completed all parts. This was usually done by a *final assessment* or *final quiz* with knowledge-based questions and test result feedback. Regarding contact with and qualification of staff, all but 1 WTP covered fewer than 30% of the 4 items in this category. Items that were addressed at least to some extent included a description of the education and qualification of the involved staff (n=3) and user contact with an HCP (partly=6). However, the latter was always only indirect contact via email or feedback forms, not live chat or Question and Answer sessions. The remaining items were not covered at all, and 7 of 15 WTPs covered none of the 4 items. Of all ratings for the abovementioned items (n=150), 29% (n=43) of the ratings were positive, and 67% (n=100) of the ratings were negative (7 rated “partly”).

### Data Security and Legal Aspects

Only 1 WTP covered 50% of the items relating to data security; another WTP covered 41%. Items that were addressed most often included *adherence to data safety* and *information regarding data storage*. All others covered 33% (n=4) or fewer (n=5) of the respective items; 4 covered none. Therefore, only 21% of all items in this category were covered on average, corresponding to a mean sum score of 2.5 out of 12. For the majority of programs, it was not clear whether user data were stored (n=10), for how long (n=13), or whether the user could request data deletion (n=12). However, 9 WTPs provided information on legal responsibilities, mostly emphasizing the user’s responsibility, for example, “The [...] shall not be responsible for any eventualities arising from the use of, or reliance on, the information contained on, or referred to in, this website or of those sites linked to this website.”

### Effectiveness, Integration Into Care, and Advertisement

WTPs covered very few items (n=5) belonging to *effectiveness*—mean sum score 0.4 out of 2; 20%, integration into care (0/4) and 27% *advertisement* (1.1/4). A total of 3 WTPs mentioned having scientifically evaluated their effectiveness, for example, on acquired factual knowledge or changes in daily activities. The rest were silent on this. Furthermore, no WTP seemed to have integrated technical interfaces for physicians or other HCPs to check the usage behavior of their patients. Finally, 8 of 15 WTPs included some form of advertisement as part of the content/learning modules, mostly related to auto-injector products for anaphylactic shock. No other WTPs explicated their avoidance of direct or indirect (hidden) advertisement. Overall, of all ratings for the 3 domains (n=75), 10 were positive (“yes”) and 64 were negative (“no”; “not included”).

### Overall Scores

On average, WTPs covered 37% of all 44 items, that is, a sum score of 33 out of 88. A total of 7 WTPs covered more than 40%, the highest score being 49% (n=1). In total, 5 covered 30% to 40% of all rated items; the rest covered fewer (n=3), the lowest score being 24% (19/88). In addition, in 13 of 15 WTPs, at least 3 domains’ respective items were not covered at all (0%). This particularly included (user) safety, contact with and qualification of staff, effectiveness, and integration into care.

## Discussion

### Principal Findings

This study aimed to analyze an exploratory sample of publicly available, free-of-charge allergy-specific WTPs regarding general characteristics, evidence base and coverage of criteria related to structure, and content and practice application. On the basis of the abovementioned aims, our main findings can be structured around the following aspects.

#### Finding Web-Based Training Programs on the Internet for Allergies

From a user perspective and as described in previous research regarding the search for WHI more generally, finding and differentiating between services appears complex [35]: first, as is evident from our own search, there seemed no particular approach that would provide a good overview of the most relevant WTPs. Instead, search results varied widely according to the search terms used. Some programs were rather found by coincidence or only when searching the website of a specific institution, which may not always be known to everyone. In addition, as selected Web-based programs varied considerably in how they named and described their specific service, understanding and comparing the different options may be difficult for nonexperts, at least regarding the different learning approaches and which of them might best suit particular user needs.

#### Domains/Items Often Covered by Web-Based Training Programs

Domains for which high(er) scores were achieved included indication (52%; 2/4), intervention (58%; 10/18), content (49%; 8/18), and user perspective (45%; 5/12). Items that were often covered as part of these domains included more general aspects, such as subject, contact details, factual information, up to dateness, and legal responsibilities. Most of these criteria may be regarded as aspects that need to be considered and communicated by any Web-based learning resource, particularly, who provides the service, what it is about, and what it aims at. In addition, providing this information may not require much (extra) effort, for example, it is straightforward to provide contact details or a funding statement.

#### Domains/Items Often not Covered by Web-Based Training Programs

The surveyed WTPs scored particularly low for the domains *effectiveness*, *qualification of staff*, *integration into care*, and *data safety* (sum scores 0%-20%). The respective criteria that were least often considered were description of education/qualification of involved staff (qualification), scientific assessment of intervention effectiveness (effectiveness), WTPs being free of barriers and available in different languages (user perspective), and details regarding data storage (data security). Even among those domains with higher sum scores, criteria, such as differentiation of symptoms (indication), intervention alternatives (intervention), differentiation of content for different target groups (user perspective), or evidence for usage time (content), were little covered. Another potential issue is the missing explanations of the choice of methods and concepts of (Web-based) learning behind the different WTP formats (see Textbox 1). Although the specific learning methods are generally clear, the reasoning behind them and adaptation to specific user groups do not become evident from WTPs’ sections, such as *About this course* or *Aims and objectives*. That this is an important shortcoming is shown by previous findings that indicate the effectiveness of asthma self-management interventions with a strong theoretical framework [36]. Apart from an WTP’s theoretical and methodological approaches, its disease-specific contents need to cover all or at least the most relevant and *right* issues, evident from latest research, medical guidelines, and expert reviews, to be of high quality. Although the assessed WTPs did provide general insights into their sources, explicit explanations regarding the content-wise completeness, for example, via systematic coverage of evidence and/or expert knowledge, were largely absent. Another aspect is the WTPs’ consideration of target groups’ (varying) levels of (digital) health literacy in the development and portrayal of methods and contents. Although the limited fulfillment of criteria related to reporting, methods, and content of health information is a barrier in its own right to improving users’ health information–related competencies, it may be important for WTPs to actively review their contents, particularly in view of users with limited health literacy, which was not apparent in this analysis. In addition, although half of the assessed WTPs addressed user satisfaction, a comprehensive and potentially in-advance assessment of users’ preferences for learning methods and potential differences among different groups may help increase WTPs’ effectiveness.

### Limitations

Our study has several limitations. First, there is no generally accepted definition of *WTP*, though the differences among the various digital formats have been described [9,37]. In addition, because of language restrictions, we included only German and English language WTPs in German. As this is an exploratory study, this approach seems to be warranted, though a broader scope would be a desirable next step, given the widespread appearance of allergies throughout the world.

Another limitation is that excluded WTPs accounted for almost half of all WTPs identified. Various WTPs could not be included, as they are currently being tested in intervention studies, or they can be accessed only via physician referral or by payment. However, such WTPs may be of higher quality; therefore, they may be more effective, which would warrant greater attention in subsequent analyses. Furthermore, our findings should not be generalized to all WTPs, that is, for other conditions or diseases. Our checklist is newly developed, used here for the first time to assess allergy-specific WTPs, and it has not yet been validated extensively. However, it relies on several well-established frameworks for developing, assessing or reporting on digital and WHI, usability, and complex behavior change interventions. Besides validating the assessment framework, it also seems important to explore users’ notions of the *quality* and *effectiveness* of WTPs, which was not enabled by our format of analysis. Finally, it was not possible to fully differentiate *no, not done* from *no, not considered* ratings in some cases. Criteria, such as information updates, may have been considered by the provider but not mentioned on the website. However, the clear and transparent reporting of these and all other aspects is crucial for maximum objectivity and comprehensibility for the user, even if some criteria may not be fulfilled for good reasons.

### Conclusions and Implications for Practice

Our findings apply to publicly available (German and English language) allergy-specific, free-of-charge WTPs. Such WTPs conform to a rather limited extent to established criteria, a shortcoming that has been noted for WHI in general [38]. Although a higher degree of conformity to quality criteria seems generally desirable, with regard to allergy-specific WTPs, a next step should be to clarify (1) what exactly an *WTP* means in the case of atopic diseases and allergies and (2) identify particularly important criteria. For instance, allergy-specific WTPs may emphasize self-management, for example, applying allergen avoidance at home, advice about treatment options (OTC drugs and specific immunotherapy), action strategies for everyday life, and help to decide when (not) to see a physician. Criteria, such as summarizing the best available evidence in lay terms and developing practical recommendations, may then be given greater weight. This suggestion is supported by our findings insofar as a number of criteria/topics were covered regularly by the majority of WTPs, although they covered others only rarely. Furthermore, the scope, target groups, and particularly methodological approaches seemed rather broad. However, for example, allergy prevention as one potential area of allergy self-management requires specific measures, and its implementation depends on patient preferences and resources. Therefore, eliciting user preferences and understanding which kinds of WTPs are deemed effective seems crucial for future practice. This current shortcoming has also been pointed out with respect to eHealth concepts more generally [20,39,40]. Better adaptation of WTPs to user preferences may finally lead to a better use of such services as part of the doctor-patient communication process [41]. Finally, none of the WTPs included made particular reference to a theoretical or pragmatic frame of reference, such as DISCERN, HON, or *JAMA*. It seems important to raise awareness of the current quality- and transparency-related shortcomings and exchange information with institutions providing WTPs—in general and specific to allergy/asthma—particularly medical associations and Public Health institutions.

## Appendix

Multimedia Appendix 1 Population, intervention, comparison, outcome table.

Multimedia Appendix 2 Assessment matrix.

Multimedia Appendix 3 Rating examples.

## Abbreviations

HCP: health care professional
HON: Health On the Net
JAMA: Journal of the American Medical Association
WHI: Web-based health information
WTP: Web-based training program
