# Supplementary material for: How Do Publicly Available Allergy-Specific Web-Based Training Programs Conform to the Established Criteria for the Reporting, Methods, and Content of Evidence-Based (Digital) Health Information and Education: Thematic Content Evaluation
Source: Interact J Med Res. 2019 Oct 24;8(4):e12225. doi: 10.2196/12225 (PMC6914270; doi:10.2196/12225)
Supplement: Multimedia Appendix 1 [file ijmr_v8i4e12225_app1.pdf]

P	I	C	O
(Population,) problem	Intervention	Comparison (Context)	Outcome
• Lay people who have one or more allergies and/or (medical) professionals making use of internet-based allergy-specific allergy training programmes	• allergy-specific online training programmes	• Comparison of selected online training programmes against each other (using sum-scores)<br><br>• Comparison of coverage of different criteria and overarching criteria-categories (44 individual criteria, 11 categories)	• Extent to which allergy-specific online training programmes cover criteria related to structure, content and quality
