# Supplementary material for: How Do Publicly Available Allergy-Specific Web-Based Training Programs Conform to the Established Criteria for the Reporting, Methods, and Content of Evidence-Based (Digital) Health Information and Education: Thematic Content Evaluation
Source: Interact J Med Res. 2019 Oct 24;8(4):e12225. doi: 10.2196/12225 (PMC6914270; doi:10.2196/12225)
Supplement: Multimedia Appendix 2 [file ijmr_v8i4e12225_app2.pdf]

	Criterion	Source	Individual rating	Group rating
**1 Indication**				
a	The symptoms addressed by the programme are described			
b	Different severity levels of the allergy for which the programme is supposed to help or not are described			
**2 Intervention**				
a	Full provider contact details are given			
b	The programme type (self-help, coaching, chat, etc.) is described			
c	The description of the type of programme is transparent and freely accessible			
d	Rationales and aims are described			
e	The programme is described separately for other target groups (who may also be interested in the content) – either for lay people or professionals			
f	A minimum/maximum usage time is mentioned			
g	A certain usage time is recommended			
h	The recommended usage time is supported by evidence			
i	Alternatives for using this particular programme are mentioned			
**3 Content**				
a	The information has been researched scientifically and systematically			
b	The information is up-to-date			
c	The information is updated regularly according to most recent available knowledge			
d	Transparent sources/references are provided			
e	The content of the information is formulated neutrally and factually			
f	The information/content points at potential uncertainties and risks			
g	Transparent information regarding financing and COI are provided			
h	Potential usage/user differences due to age or sex are mentioned			
i	The content is differentiated/adapted for			

	different target groups			
**4 Safety**				
a	Potential unintended effects due to using the programme are described			
b	The programme describes the systems' reaction towards unintended effects			
**5 Qualification**				
a	Users can contact an expert			
b	The qualification of the expert is described (if they are part of the intervention)			
c	Experts that can be contacted use an intervention manual			
d	Experts are being supervised			
**6a Effectiveness**				
a	The effectiveness of the programme is assessed (via a scientific evaluation)			
**6b User perspective**				
a	The programme is free of barriers (e.g. hearing or vision impairment of users)			
b	The programme is free of charge			
c	The programme is available in different languages			
d	Completion/termination rates are mentioned			
e	User satisfaction with the programme is assessed			
f	The success of the programme is assessed (have the users completed the modules etc. successfully)			
**7 Integration into care**				
a	User behaviour is followed up			
b	Users can exchange/communicate with other users and/or other professionals as part of the programme			
**8 Legal aspects**				
a	There is a description of who is liable in case of mistakes, adverse effects, etc.			
**9 Data safety**				
a	It is clearly mentioned that data safety is ensured by the provider			
b	The user can register anonymously			
c	User data are stored			
d	It is mentioned for how long the data are stored			
e	The user can ask the provider to delete personal data			

f	The programme requires a specific IT system			
**10 Advertisement**				
g	The programme includes open/direct advertisement			
h	The programme included indirect advertisement			
