# Supplementary material for: How Do Publicly Available Allergy-Specific Web-Based Training Programs Conform to the Established Criteria for the Reporting, Methods, and Content of Evidence-Based (Digital) Health Information and Education: Thematic Content Evaluation
Source: Interact J Med Res. 2019 Oct 24;8(4):e12225. doi: 10.2196/12225 (PMC6914270; doi:10.2196/12225)
Supplement: Multimedia Appendix 3 [file ijmr_v8i4e12225_app3.pdf]

Criterion	Source	Text example	Rating
**Indication**			
1a Symptoms	ISW	It can make the skin dry, itchy, red, broken and sore. It sometimes makes the skin darker or lighter for a while.	Yes
1b Symptom levels	Bist du auch allergisch?	Die Online-Schulung bietet den Erzieher/innen und Pädagogen die Möglichkeit, sich das Thema „Allergie” eigenständig online zu erarbeiten.	No
**Intervention**			
2 Contact details	Asthma Management and Education Course	For more information or assistance please contact Liana Burns, Educational Programs Manager, at 202-466-7643 ext. 252 or liana@aafa.org	Yes
	ISW	INTEGRATED RESPIRATORY & ALLERGY CARE PATHWAY PROJECT	Partly
	Allergyaware	The Allergy Aware website and courses have been developed by Food Allergy Canada (formerly Anaphylaxis Canada) and Leap Learning Technologies Inc., in partnership with the Canadian Society of Allergy and Clinical Immunology	Partly
3a Time recommendation	Asthma Basics	How long will it take me to complete this course?<br>This course is estimated to be completed within 1 - 2 hours.<br>Do I have to complete this course all at one time?<br>The course can be completed at your own pace. If you can't complete the course the same day, your progress and completed activities will be stored and saved for the next time you come back to the course.	Yes
3b Time recommendation evidence	ISW	The pathways have undergone an exhaustive approval process and are now ready for implementation.	Partly
**Content**			
4 Systematic research	NACA	The information and treatment protocols presented on the National Asthma Council website such as the handbooks, pamphlets and educational materials are based on current medical knowledge and practice as at the date of publication.<br><br>References specific to each section of this module will be supplied, but the source of much of the	Yes

		information is the Australian Asthma Handbook, the best-practice, evidence-based national guide for primary health care professionals.	
	Bist du auch allergisch?	Die Zusammenstellung der Informationen erfolgte mit der gebotenen Sorgfalt. Gleichwohl übernehmen wir keinerlei Haftung, aus welchem Rechtsgrund auch immer, für die Richtigkeit, Aktualität und Vollständigkeit der übermittelten Informationen.	Partly
	Itchy S W	Allergy is the word used to describe a bad reaction that the body has to a particular substance in the environment. Most substances that cause allergies are not harmful and have no effect on people who are not allergic.<br>[…]<br>Adapted from:<br>http://www.nhs.uk/conditions/Allergies/Pages/Introduction.aspx	Partly
5 Sources	WAO	Asthma and Allergic Rhinitis: WAO Online Lecture Series Module 1. The underlying mechanisms of the “United Airway” concept Authors: Motohiro Ebisawa, MD, PhD; Ruby Pawankar, MD, PhD Sources/Links Beasley R; The International Study of Asthma and Allergies in Childhood (ISAAC) Steering Committee, Worldwide variation in prevalence of symptoms of asthma, allergic rhinoconjunctivitis, and atopic eczema: ISAAC. Lancet 1998; 351(9111): 1225-1232. (Study of worldwide prevalence of atopic diseases in 463,801 children 13–14 years of age. Children self-reported symptoms over 12 months using questionnaires) Summary Bousquet J, Anessi Maesano I, Carat F et al, Characteristics of intermittent and persistent allergic rhinitis: DREAMS study group. Clinical and Experimental Allergy 2005; 35(6): 728-732. Abstract Bousquet J, Khaltaev N, Cruz AA et al, Allergic Rhinitis and its Impact on Asthma (ARIA) 2008 Update (in collaboration with the World Health Organization, GA2LEN* and AllerGen**) Allergy. 2008;63(suppl 86):8–160. Full text<br>[…]	Yes
	Allergyaware	Some content for this course was adapted with permission from the consensus guidelines, Anaphylaxis in Schools & Other Settings, 3rd Edition Revised. © 2005–2016 Canadian Society of Allergy and Clinical Immunology.	Partly

6 Up-datedness / regular update	Allergy and anaphylaxis e-training for HCP	ASCIA will retain copyright and control over content of ASCIA allergic rhinitis e-training HP, and update it as evidence based changes occur in the medical literature.; Last modified: Tuesday, 17 November 2015, 11:39 AM	Yes
	Bist du auch allergisch?	Die Zusammenstellung der Informationen erfolgte mit der gebotenen Sorgfalt. Gleichwohl übernehmen wir keinerlei Haftung, aus welchem Rechtsgrund auch immer, für die Richtigkeit, Aktualität und Vollständigkeit der übermittelten Informationen.	Partly
	Itchy S W	Funding from the National Institute for Health Research (NIHR) Collaboration for Leadership in Applied Health Research Care (CLAHRC) for North West London has been secured for the implementation of the integrated care pathways over the next 18 months within Hammersmith and Fulham, Kensington and Chelsea and Westminster. We have developed a project board and are now recruiting GP practices in which to commence the programme.	Partly
7 Conflict of Interest, funding	ASCIA	The ASCIA website is directly funded by ASCIA and therefore ASCIA does not seek nor receive any educational grants to fund this website. Hence there are no conflicts of interest regarding the sources of funding and the editorial content of the website.	Yes
	WAO	Sponsored by an unrestricted educational grant from Merck, Inc.	Partly
**Qualification of staff**			
8 Contact to staff	Asthma management and education course	For more information or assistance please contact Liana Burns, Educational Programs Manager, at 202-466-7643 ext. 252 or liana@aafa.org // Please leave a message including name, number and your issue. We will return your call within 1 hour.	Partly
	Human biomonitoring	Spezielle Fragen zum Human-Biomonitoring beantworten die Autoren, oder sie leiten die Frage zur Beantwortung weiter (z. B. an die Informationsstelle Human-Biomonitoring).	Partly
		Meet the Experts: Our multidisciplinary team of health professionals, advocates, researchers and educators brings you the best education.<br><br>[List of individuals without contact options]	No
**User perspective**			

9 Effectiveness, evaluation	Allergy ready	In a research study of our program with over 70 school personnel it was proven to be highly effective, with large improvements in knowledge, skills and attitudes after using the course.	Yes
	Allergy and anaphylaxis e-training for HCP	Completing this short evaluation allows ASCIA to ensure this course meets the requirements of general practitioners and paediatricians in managing patients with allergy and anaphylaxis and allows ASCIA to provide this feedback to the RACGP as part of the reporting process	Yes
	Asthma Basics	Knowledge and satisfaction questionnaire 90-day follow up	Partly
**Data safety**			
10 Data storage	Allergyaware	A: In order to provide participating users with a Certificate of Completion for Online Courses, your activities on the Website such as viewing e-learning module screens or responding to quiz questions are recorded in a secure database. In addition to the registration process, as part of your use of Website or participation in the Online Courses, we may obtain certain information about you and your performance in the Online Courses. Some of this information may be personally identifiable information. We may use, maintain, and store this information to provide certain services to you now and in the future.<br>B: Some of this information may be personally identifiable information. We may use, maintain, and store this information to provide certain services to you now and in the future. For example, the ability for you to generate and download a Certificate of Completion based on Online Course module requirements.	Yes
	e-learning hub	Your progress will be recorded and once all modules have been undertaken successfully, you will be emailed a certificate of completion.	Partly
11 Data deletion	NACA	If you want to change any information that you have previously given us, or if you want to opt out of future communications please contact us.	Partly
	Azerta	Dateschutz: Entsprechend der gesetzlichen Vorschriften wird auf das Recht des Nutzers auf unentgeltliche Auskunft über die gespeicherten Daten, auf Löschung der gespeicherten Daten sowie auf das Widerspruchsrecht zur Erstellung und Verwendung seines anonymisierten Nutzungsprofils hingewiesen.	Yes
